# An SUI-based approach to explore visual search results cluster-graphs

**DOI:** 10.1371/journal.pone.0280400

**Published:** 2023-01-20

**Authors:** Umer Rashid, Maha Saddal, Ghazanfar Farooq, Muazzam Ali Khan, Naveed Ahmad

**Affiliations:** 1 Department of Computer Science, Quaid-i-Azam University, Islamabad, Pakistan; 2 Department of Computer Science, Prince Sultan University, Riyadh, Saudi Arabia; Shandong Normal University, CHINA

## Abstract

Nowadays, exponential growth in online production and extensive perceptual power of visual contents (i.e., images) complicate the users’ information needs. The research has shown that users are interested in satisfying their visual information needs by accessing the image objects. However, the exploration of images via existing search engines is challenging. Mainly, existing search engines employ linear lists or grid layouts, sorted in descending order of relevancy to the user’s query to present the image results, which hinders image exploration via multiple information modalities associated with them. Furthermore, results at lower-ranking positions are cumbersome to reach. This research proposed a Search User Interface (SUI) approach to instantiate the non-linear reachability of the image results by enabling interactive exploration and visualization options. We represent the results in a cluster-graph data model, where the nodes represent images and the edges are multimodal similarity relationships. The results in clusters are reachable via multimodal similarity relationships. We instantiated the proposed approach over a real dataset of images and evaluated it via multiple types of usability tests and behavioral analysis techniques. The usability testing reveals good satisfaction (76.83%) and usability (83.73%) scores.

## Introduction

Recent years witnessed an exponential increase in the online exploration of visual content [1]. Humans are proficient in recognizing visual patterns instead of recalling non-visual contents [2]. They prefer to recognize the information objects in information search and exploration-related activities [3]. However, the visual content, especially image objects, is aggregated with textual information such as annotations, tags, descriptions, etc., which enhances the humans’ information-seeking activities [4]. Using images and related textual information over the web enhances user comprehension [5]. The image exploration services over social media platforms such as twitter (https://twitter.com/), Facebook (https://www.facebook.com/), and Instagram (https://www.instagram.com/), etc., also show advancements in the recent years [6–8].

Web users generally interact with image-related visual information in finding, exploration, and discovery-related search activities [9]. Images engagement in web search and users’ exploration activities is higher than contents belonging to other media formats [10]. The web search engines provide retrieval mechanisms to satisfy the visual information needs [11]. The users may express their information needs as textual query terms and interact with the visual contents [12]. However, the increase in imaginary information and retrieval via web search engines makes exploration services challenging [9]. The information needs are profoundly affected triad of visual retrieval, which requires intriguing advance and comprehensive lookup, exploration, and discovery mechanisms in web search [11, 13, 14].

The recent research emphasizes image exploration by intriguing exploration and visualization in SUIs. Therefore, the current study addresses two main issues, i.e., results representation and exploration via interactive SUIs. Firstly, to identify relationships in results and represent them by exploiting primitive data models, e.g., lists, graphs, trees, etc. Secondly, to enable the exploration of results via various interactive exploration possibilities. The data model provides a baseline or an arrangement to explore the search results interactively. In addition to this, the data models offer particular types of relationships in the search results that may enhance the users’ exploration activities. The SUIs are usually specific to data models and allow individual interaction with results by exploiting advanced information presentation and visualization components. We address the latter issue in this research. Our previous study also addressed the architectural and data modeling problems concerning the image results exploration [9, 15].

### Problem statement

**Fig 1:**
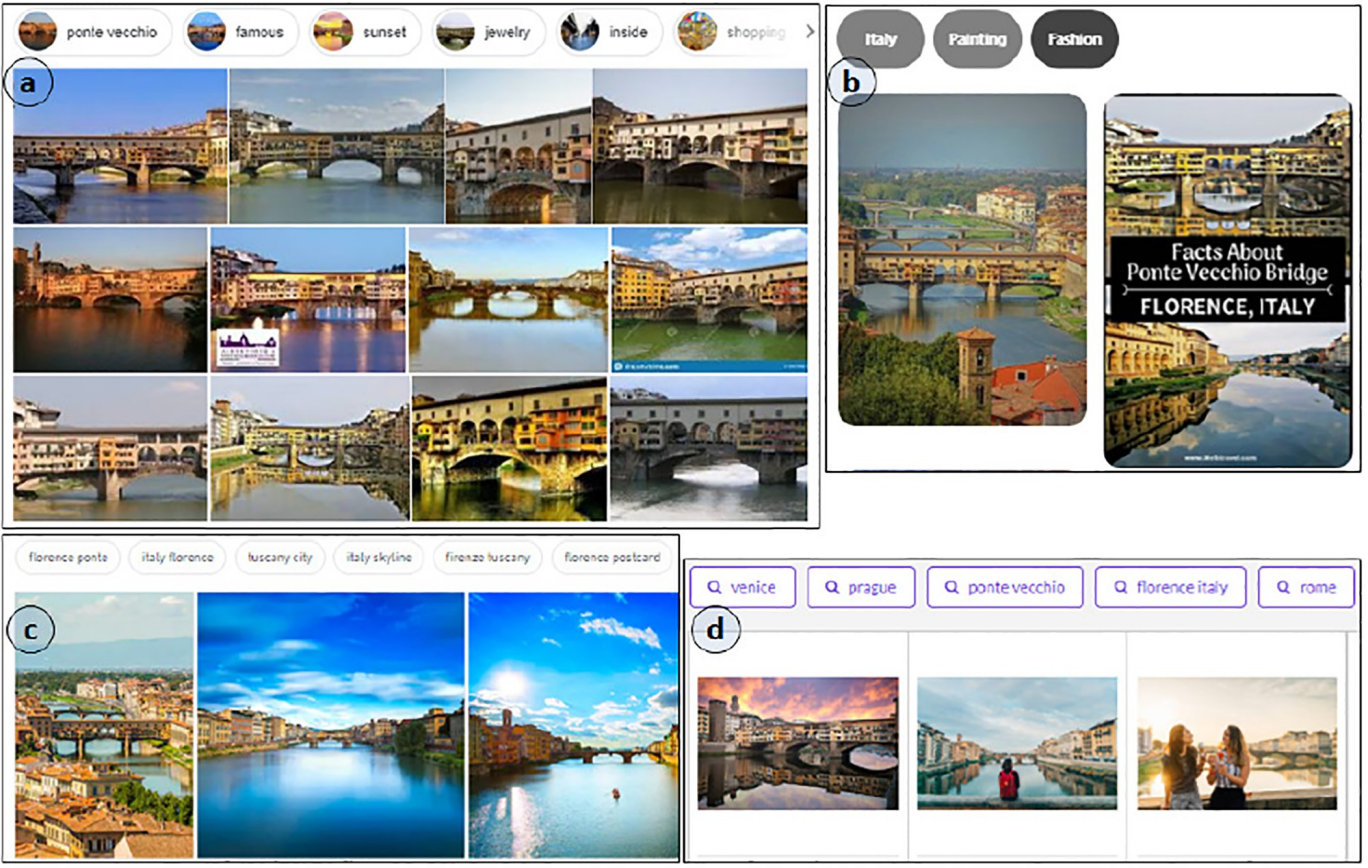
Presentation of image results over web search engines in semantically relevant titles/clusters.

The SUI exploration approaches are based on linear data models and primarily employ ranking algorithms that present the results in graphical panels to enable lookup-based interactions. In the lookup scenarios, exploration activities are linear, which involves linearly accessing results by traversing images in lists or grids. The exploration involves searching, navigating, accessing, and interacting with the web image results. However, the results at lower-ranking positions are not reachable during lookup activities. Furthermore, the relationships among the search results are unknown, except for their proximal ranking positions. The web search engines, for example, Google (https://www.google.com/), Shutterstock (https://www.shutterstock.com/), Gettyimages (https://www.gettyimages.com/), and Picinterest (https://www.pinterest.com/), etc., provides the mentioned interaction with image results (Fig 1). Alternatively, non-linear data models usually organize the results in graph or tree representations by exploiting mono-modal similarity or semantic relationships to connect the images and enable navigation and in-depth browsing. The graphs and clustering approaches are usually not combined to provide the reachability of desired graph regions. They lack a comprehensive focus on the issues associated with navigation, and reachability arises due to the representation of results in dense graphs. The user navigation within results to reach desired results is cumbersome.

This research aims to address the users’ image exploration issues. Therefore, we investigated an SUI-based approach to explore and visualize the image results. The SUI augments exploration activity via the underlying instantiated cluster-graph data model to represent associated results. The users can explore image results via an SUI design by selecting relevant clusters and navigating them to satisfy their visual information needs. Mainly, this research represents the image results via a cluster-graph and enables their exploration via a Search User Interface (SUI) design. The objective is to address reachability issues associated with image exploration and provide navigation and browsing images within cluster-graphs of results via interactive SUI components.

### Contributions

As a preliminary investigation, a few concepts related to the non-linear exploration of images have been presented in our previous research [9]. We further emphasized architectural, graph-data model, and algorithmic aspects in another study [15]. On the contrary, in this research, we focused a comprehensive SUI approach to explore and visualize images in an integrated way. We initially represented images as graph nodes and multimodal similarities as edges. We converted the graphs into forests containing clusters of relevant and connected results. We provided an interactive SUI to make the exploration convenient by including visualization and access to images represented in a cluster-graph layout. This research investigates a comprehensive SUI approach to explore and visualize images in an interactive, integrated, and usable way. Notably, in this research, we:

- presents a complete formalization of an SUI approach.
- utilizes a real dataset of images containing accompanying textual contents and comprehensive visual features.
- provides a complete instantiation of the exploration approach.
- implements a full-fledged tool and associated SUI.
- employs multiple types of tests to evaluate the usability of SUI and associated exploration scenarios.

Over investigation reveals that the successfully-task completion ratio was high, the users’ exploration experience was satisfactory, and the task difficulty rating was low in all user groups. The respondents preferred the advanced interaction mechanism in image results exploration via cluster-graphs. The System Usability Scale (SUS) and Computer System Usability Questionnaire (CSUQ) evaluation also give reasonably good satisfaction (76.83%) and usability (83.73%) scores.

## Literature review

### Background

The web search engines provide a comprehensive presentation of image results [15]. The exploration involves interaction with results presented via linear lists or grid layouts. The linear exploration mainly includes horizontal-vertical traversing [16]. The users can overview, preview, view, and access results. The ranking decisions affect the reachability since lower-ranked results are usually not accessible [17]. Recently, web image search engines, e.g., Google, Shutterstock, Gettyimages, Picinterest, etc., provide titles to interact with results (Fig 1). The objective is to reduce the exploration complexity by title-specific filtering of images. However, the presentation is grid-based; the interaction is still linear; resulting in lower-ranking positions not being non-linearly reachable.

Recent research studies emphasize image exploration needed by intriguing exploration and visualization in SUI design [18]. Information retrieval is mainly concerned with two issues; results representation and investigation via an interactive SUI design. The former identifies relationships in results and represents them by exploiting primitive data models, e.g., lists, graphs, trees, etc. The latter enables the exploration of results via various types of interactive exploration possibilities [19, 20]. The SUIs are usually specific to data models and allow particular interaction with results. The research proposed in this paper generally addresses later issues in image results exploration over the web.

### Exploration approaches

Web search engines usually explore images via ranked lists or grid layouts. Gary Marchionini introduced exploratory search by interrelating faceted browsing [21]. Capra and Marchionini recommended facets of semantically related search results to provide topic-specific facets access linearly [22]. Marti A. Hearst interrelated the terms browsing, navigation, and visualization via SUIs [23]. Rashid et al. introduced the exploration of search result space instantiated via graph data models and SUIs [12, 24]. In recent years, state-of-the-art tools/approaches have been suggested to provide non-linear exploration of visual content; e.g., ISRE-Framework [15], MIRRE-Approach [5], M^2^IS-Tool [24], FACETS [25], Media Finder [26], Image Search interface [27] etc.

### Non-linear data models

In advanced exploration mechanisms, the non-linear representations like graphs and trees connect search results via non-linear relationships [11, 12, 28]. In non-linear representations, the nodes represent visual contents connected via different types of relationships as edges [5]. A tree is also a restricted form of a graph depicting a hierarchical structure containing minimal connected components [29]. The trees may convert into forests or cluster-graphs by removing irrelevant edges. The cluster- graph represents multiple clusters of search results and reduces graph complexity [9, 15]. Generally, non-linear representations enable in-depth browsing of search results that can allow users to navigate and reach results non-linearly by exploiting multiple types of relationships [24, 30]. At the base of non-linear data models, the exploration tools provide access to integrated retrieved contents in a usable way.

### SUI-based exploration mechanisms/tools

ISRE-Framework elaborated architectural and algorithmic aspects associated with exploring web image results. The framework provides the discovery of web image results effectively by organizing them in cluster-graph data models [15]. The MIRRE-Approach enabled the exploration of multimedia documents by encapsulating the textual and visual content via a graph data model and an interactive SUI. The approach also provides a tool representing multimedia documents as a search result space of non-linearly connected multimedia content. The tools enable non-linear interaction with multimedia document results via interactive exploration options [5]. The *M*^2^*IS*-Tool empowered the exploration of multimedia contents, including images, via a graph data model and an interactive GUI. The tool represents multiple media formats, such as a non-linear search result space, and enables non-linear interaction with them via interactive exploration options [24]. The FACETS tool visualized millions of graph nodes as image results in facets. The approach guides users in exploring desired nodes and neighbors in facets non-linearly [25]. The image Search interface suggested by Hoque et al. provides the visualization and interaction techniques for exploring and refining query terms via multiple presentations. The grid-based visualization enables users to pan and zoom through results. The tree view organizes query-related concepts and maintains query history. The concept hierarchy presents conceptual categories via a tree structure [27]. The Media Finder interacted with related image content available over multiple social media networks. The standard document model of the tool aligns results retrieved through various sites non-linearly by employing textual modality. The non-linearly categorized grid layout presents image results [26]. Visual Islands enabled the exploration of multimedia news containing images. The tool clusters news contents in visual islands that are navigatable by the users [31]. Faeric World provides searching, browsing, and visualization of image contents by a graph data model. The thematic and reference relationships provide navigation within the image search results. The graph contains links to image results; however, the images in clusters represent textual similarity in them [32].

## Exploration approach

### Overview

The proposed Search User Interface (SUI) approach provides the nonlinear reachability of image results via interactive exploration and visualization options. Users with exploration needs interact with SUI via queries. The approach utilizes a cluster-graph data model to represent image objects as nodes and multimodal similarity relationships as edges. Image results in cluster-graph are non-linearly reachable. The users can look up, explore, and navigate the image result clusters in an integrated way.

### Preliminaries

**Fig 2:**
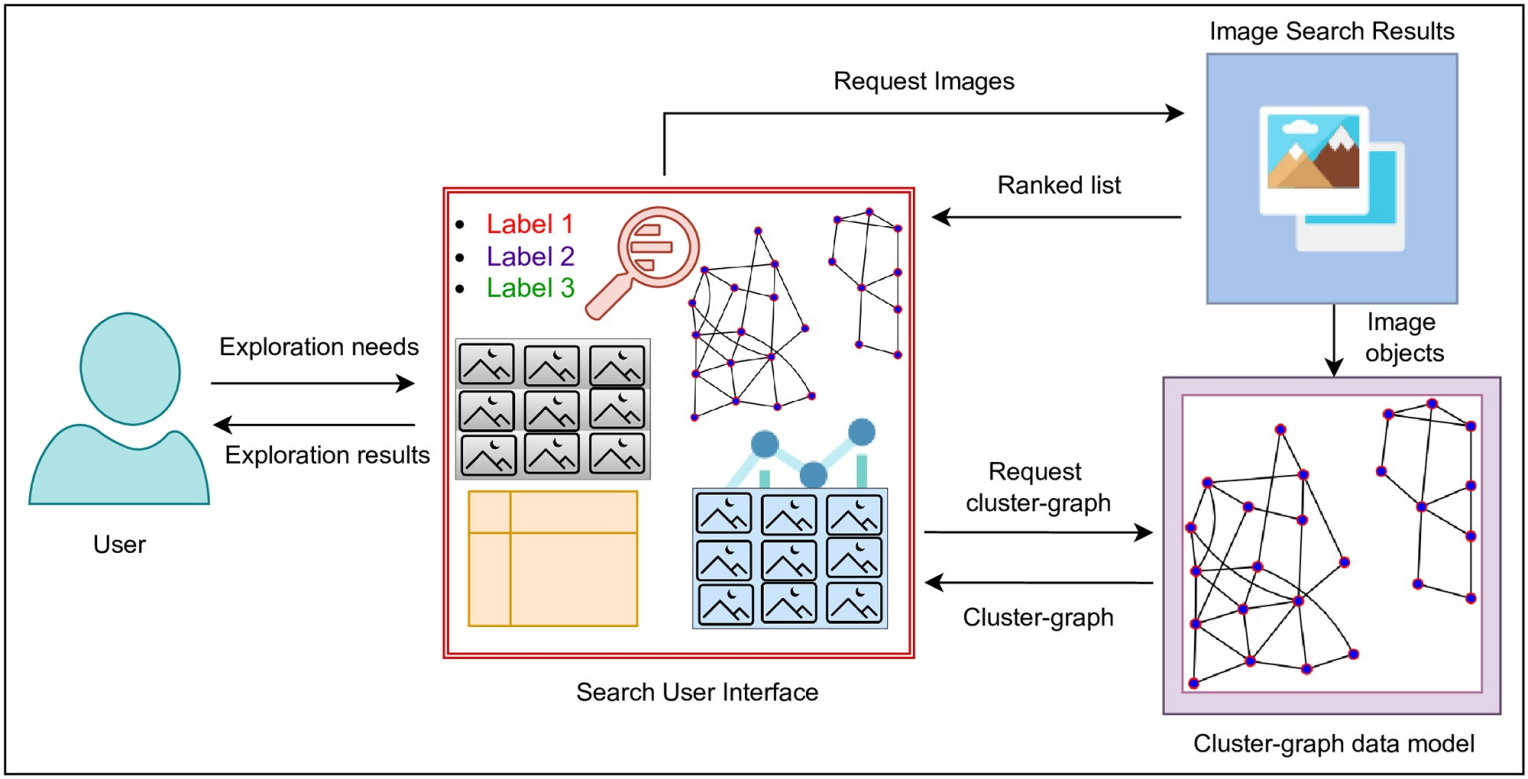
An overview of the proposed approach.

The proposed approach exploits multimodal information associated with images to construct a cluster-graph data model and enable exploration via an SUI design. The former instantiates a non-linear multimodal result space over images and later exploits a cluster-graph result space in exploration and visualization. The objective is to enable image retrieval against textual query terms, instantiate a cluster-graph data model over images, and allow the exploration and visualization of images via an interactive SUI design. Our approach mainly contains a cluster-graph data model and an SUI design. Fig 2 depicts our approach.

### Formalization

**Fig 3:**
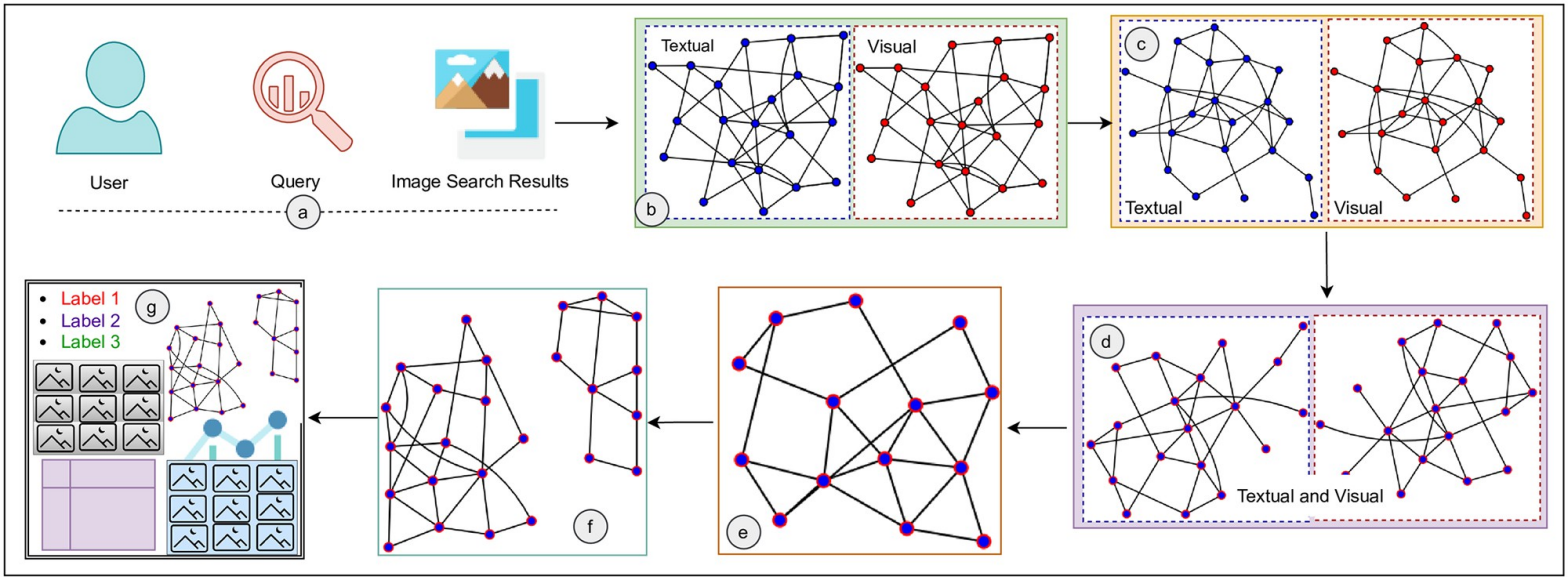
Schematic representation of approach architecture depicting; Image results retrieval (a), Dense and complex graphs (b), Simplified image graphs (c), Converted image similarity graphs (d), Multimodal image tree (e), Image forest (f), and Exploration components (g).

We retrieved images (Fig 3(a)), and constructed labeled weighted Textual and visual Mesh Graphs *T*_*GM*_ = (*V*, *E*_*t*_) and *V*_*MG*_ = (*V*, *E*_*v*_), respectively (Fig 3(b)). In *T*_*MG*_ and *V*_*MG*_ nodes *V* = {*n*_1_, *n*_2_, …, *n*_*m*_} are images, particularly:

$$\begin{array}{c} E=\{\left\{E_{t}\right\}\cup \left\{E_{v}\right\}\} \end{array}$$ (1)

where $E_{t}=\{E_{t_{1}},E_{t_{2}},...,E_{t_{n}}\}$ and $E_{v}=\{E_{v_{1}},E_{v_{2}},...,E_{v_{n}}\}$ are weighted sets of edges representing textual and visual similarities in nodes respectively. In *T*_*MG*_, the weight *w*_*tk*_ of an edge *e*_*tk*_ incident on a pair (*n*_*i*_, *n*_*j*_) represents textual similarity of keywords associated with *n*_*i*_ and *n*_*j*_, where *e*_*tk*_ ∈ *E*_*t*_ and *n*_*i*_, *n*_*j*_ ∈ *N*. Similarly, in *V*_*MG*_, weight *w*_*vk*_ of an edge *e*_*vk*_ incident on a node pair (*n*_*i*_, *n*_*j*_) represents the visual distance of descriptors associated with *n*_*i*_ and *n*_*j*_, where *e*_*vk*_ ∈ *E*_*v*_, and *n*_*i*_, *n*_*j*_ ∈ *N*.

Our approach minimizes *T*_*MG*_ and *V*_*MG*_ complexity by removing edges having weights greater than a certain threshold value (Fig 3(c)). We normalized the edge weights of graphs by converting the distance edge weights of *V*_*MG*_ into similarity edge weights (Fig 3(d)). Our approach unifies *T*_*MG*_ and *V*_*MG*_ to instantiate a Union Multimodal Graph *U*_*MG*_ = (*V*, *E*_*m*_), since both graphs have same set of nodes, but a different set of edges, therefore:

$$\begin{array}{c} E_{m}=\{\left\{E_{t}\right\}\cap \left\{E_{v}\right\}\} \end{array}$$ (2)

where $E_{m}=\{E_{m_{1}},E_{m_{2}},...,E_{m_{n}}\}$ (Fig 3(e)). In *U*_*MG*_, an edge *e*_*mi*_ connects a pair (*n*_*i*_, *n*_*j*_), if and only if connected via *e*_*ti*_ and *e*_*vi*_ in their corresponding *T*_*MG*_ and *V*_*MG*_ respectively; where *n*_*i*_, *n*_*j*_ ∈ *N*, *e*_*mi*_ ∈ *E*_*m*_, *e*_*ti*_ ∈ *E*_*t*_, and *e*_*vi*_ ∈ *E*_*v*_. Our approach finally converted *U*_*MG*_ into a disjoint trees union (cluster-graph) and employ it in non-linear exploration and visualization via an interactive SUI design (Fig 3(f) & 3(g)).

### Cluster-graph data model

The cluster-graph data model is represented by an acyclic, weighted labeled graph *U*_*MG*_ = (*V*, *E*), where *V* and *E* represents set of nodes and edges respectively. The results denoted by *p*_*i*_ are nodes in *V* = {*n*_1_, *n*_2_, *n*_3_, …, *n*_*m*_} and *t*_*i*_ are image titles displayed over nodes as captions. ∀*e*_*i*_ ∈ *E*, where *E* = {*e*_1_, *e*_2_, *e*_3_, …, *e*_*n*_}, has corresponding non-negative weights in set:

$$\begin{array}{c} w\left(E_{n}\right)=\{w_{1},w_{2},w_{3},...,w_{k}\} \end{array}$$ (3)

where, *w*(*E*_*n*_) are multimodal similarity relationships between images. The set *E* contains multimodal links *E*_*m*_. The labels of multimodal edges are similarity weights. There is no compulsion that ∀*n*_*m*_∃*e*_*n*_, since there is possibility of nodes having no edge at all. The degree of a node *deg*(*V*) is number of edges incident on it.

In *U*_*MG*_, ∀*e*_*i*_ ∈ *E*_*m*_, *deg*(*V*_*i*_) ≥ 1, because in presence of edges there is an association in image nodes. Alternatively, ∀*e*_*i*_ ∉ *E*_*m*_, *deg*(*V*_*i*_) = 0, because in absence of edges there is no association in image nodes. The *U*_*MG*_ contains a group of sub-graphs called clusters *C*, where *C* = {*c*_1_, *c*_2_, *c*_3_…, *c*_*n*_}. ∀*c*_*i*_ ∈ *C*, ∣*c*_*i*_∣ ≥ 1, since each cluster *c*_*i*_ contain one or more images connected via multimodal similarity edges. ∀*c*_*i*_ ∈ *C* contains a sub-graph of *U*_*MG*_ and a set of sub-graph $U_{MG}^{\prime }$ may contains multiple sub-graphs, represented as:

$$\begin{array}{c} U_{MG}^{\prime }=\{U_{MG_{1}}^{\prime },U_{MG_{2}}^{\prime },...,U_{MG_{n}}^{\prime }\}∵U_{MG}^{\prime }\in U_{MG} \end{array}$$ (4)

### SUI design

**Fig 4:**
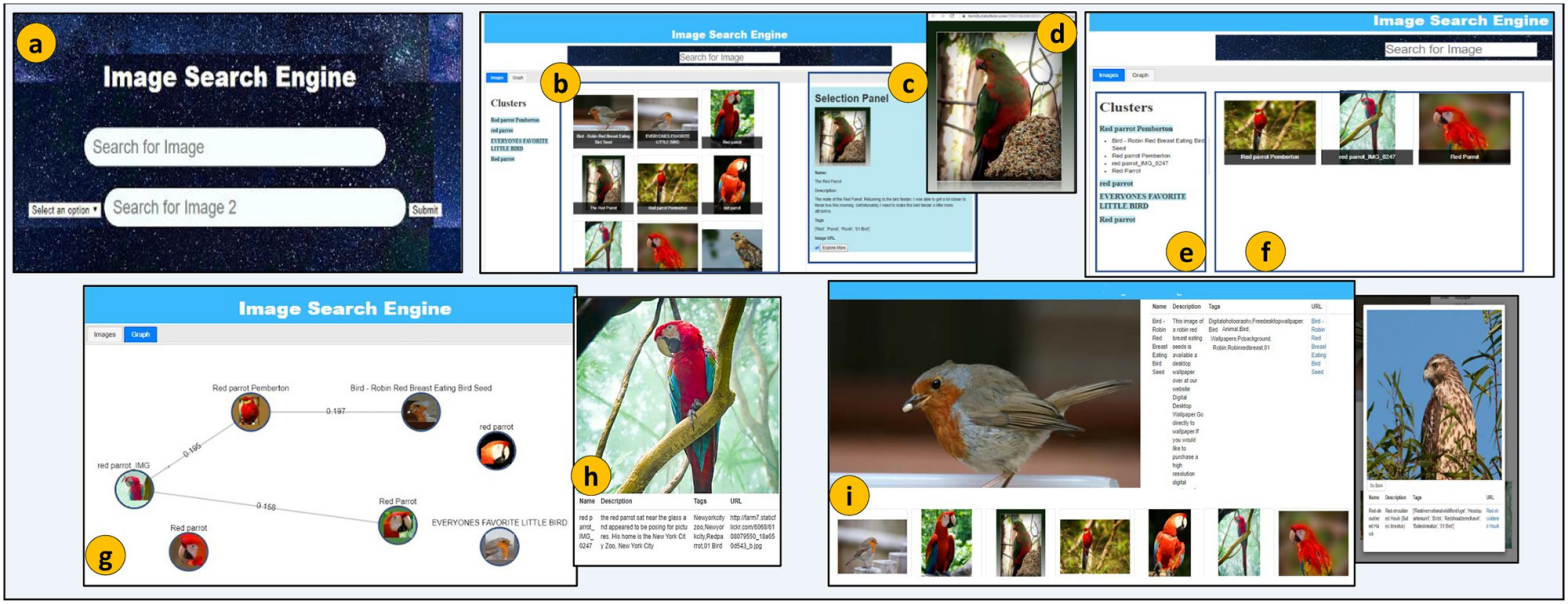
Query Formulation Panel (QFP) (*a*), Result Grid Panel (RGP) (*b*), Selection Panel (SP) (*c*), access of image result from the actual web source (*d*), Cluster List Panel (CLP) (*e*), Cluster-Graph Panel (CGP) (*f*) Graph Visualization Panel (GVP) (*g*), Information Window (*h*), and Related Results Panel (RRP) (*i*) of SUI.

The SUI design contains multiple panels to provide interaction with images. Notably, SUI design focuses on the in-depth browsing of clusters. The objective is to (i) reach relevant clusters within a cluster-graph and (ii) access desired images within clusters in a non-linear and multimodal way (Fig 4).

The QFP allows the expression of information needs via keyword-based Boolean queries (Fig 4(a)). RGP presents results in a 2-D grid layout containing image thumbnails and textual captions in a ‘standard grid’. The users can overview first and traverse horizontally-vertically the ranked images (Fig 4(b)). The SP provides a detailed view of user-selected results from other panels (Fig 4(c)). The users can select any image from RGP, CLP, CGP, or GVP to view associated details. The view includes the image thumbnail, title, description, user tags, and URI of a selected result. The users can also access chosen images from their actual web sources (Fig 4(d)).

The CLP presents the instantiated cluster-graph as clusters. In CLP, each cluster contains similar images connected via multimodal similarity relationships. The CLP visualizes textual representatives of clusters as labels. The CLP provides extendable hierarchies of images in a cluster-graph. The extendable cluster items provide the filtering of results. The users can select a cluster of their interest and browse the image results (Fig 4(e)). The CGP presents ranked clusters via a dynamic and interactive 2D grid. The image thumbnails and their associated textual captions visualize results. The CGP presents ranked results in a user-selected cluster. The ranking is performed by considering the textual similarity of the user-selected image with the rest of the results in CGP. This panel provides in-depth browsing of images, where users can select results and view their directly connected images. The CGP enables navigation or in-depth browsing of clusters (Fig 4(f)).

The GVP visualizes the cluster-graph instantiated over results in a dynamic graph containing disconnected components called clusters. The nodes visualize images as thumbnails in the graph and edges as multimodal similarity relationships. In GVP, the users can preview images in the form of a cluster-graph, zoom results, drag nodes, navigate within clusters, select desired results, and view details of selected images in an integrated way by following the basic visualization mantra (Fig 4(g)). The users can choose a result from SUI panels and view related details. The RRP visualizes neighbors of results in SP. The RRP previews related images and shows ranked results as thumbnails without their associated captions in a horizontal strip. The users can view associated textual information by selecting an image from RRP and can locate the results in RGP and CGP (Fig 4(i)).

## Instantiation

We instantiated the proposed approach on a real dataset of images retrieved from online sources. However, the approach is generic and applicable to other datasets of images. We developed a tool to enable the non-linear and multimodal exploration of images retrieved against textual query terms. We used Python (https://www.python.org/) programming language to develop the tool in PyCharm (https://www.jetbrains.com/pycharm/download/) Professional integrated development environment (IDE). Furthermore, Django (https://www.djangoproject.com/) web framework was used to integrate the several exploration components. The dataset, image results exploration tool, video tutorial, source code, installation readme, and evaluation data are freely available at Zenodo repository (https://doi.org/10.5281/zenodo.6388246/).

### The dataset & descriptors extraction

We employed the I-Search dataset (https://vcl.iti.gr/dataset/i-search-multimodal-dataset/) containing more than 10K instances of images in XML format retrieved from Flickr (https://www.flickr.com/) server. There exist 51 categories in the dataset where each category has approximately 200 images. The XML-based representation includes images associated with the creator, title, description, user tags, URIs, etc. The instances in the dataset are sufficient since multiple studies used the same dataset to instantiate exploration approaches [12, 24, 33]. We extracted textual and visual descriptors from the images. The former is associated with images as accompanying text (i.e., titles, textual descriptions, user tags, etc.) and extracted as keywords via a routine in *C*#. The latter is associated with images as Color and Edge Directivity Descriptors (CEDD) and extracted via CEDD Library (https://chatzichristofis.info/?page_id=15). The CEDD descriptors consider edge and color information and are recommended in the literature for visual approximations, which are less complicated and give accurate approximations [34, 35]. The descriptors are extracted and stored in Comma-Separated Values (CSV) files.

### Indexing & retrieval

We instantiated inverted index of images over the textual descriptor stored in CSV files via Elasticsearch (https://lucene.apache.org/), Lucene (https://lucene.apache.org/), and RESTful [36] web services, all are used to instantiate document-oriented inverted indexes. Initial pre-processing such as stop-word removal and stemming, was performed to clean the data. Afterward, we implemented the indexing in Python using Elasticsearch and Lucene libraries. The inverted index maintained keywords as vocabulary terms and images in postings. In postings, we also maintained pointers to CEDD (stored in separate CSV files). We retrieved images by employing Elasticsearch to handle a wide range of queries and enable multi-tenant textual retrieval [36]. The query terms are parsed and processed via Elastic-search Query Parser. The Elasticserch can build complex Boolean queries by utilizing Query-DSL (JSON-based query), which allows the formulation of keyword-based query fields comprising OR, AND, and NOT clauses. In retrieval, Elasticsearch matches query terms with tokens and ranks image results via a vector space model [37].

### Graph instantiation

**Fig 5:**
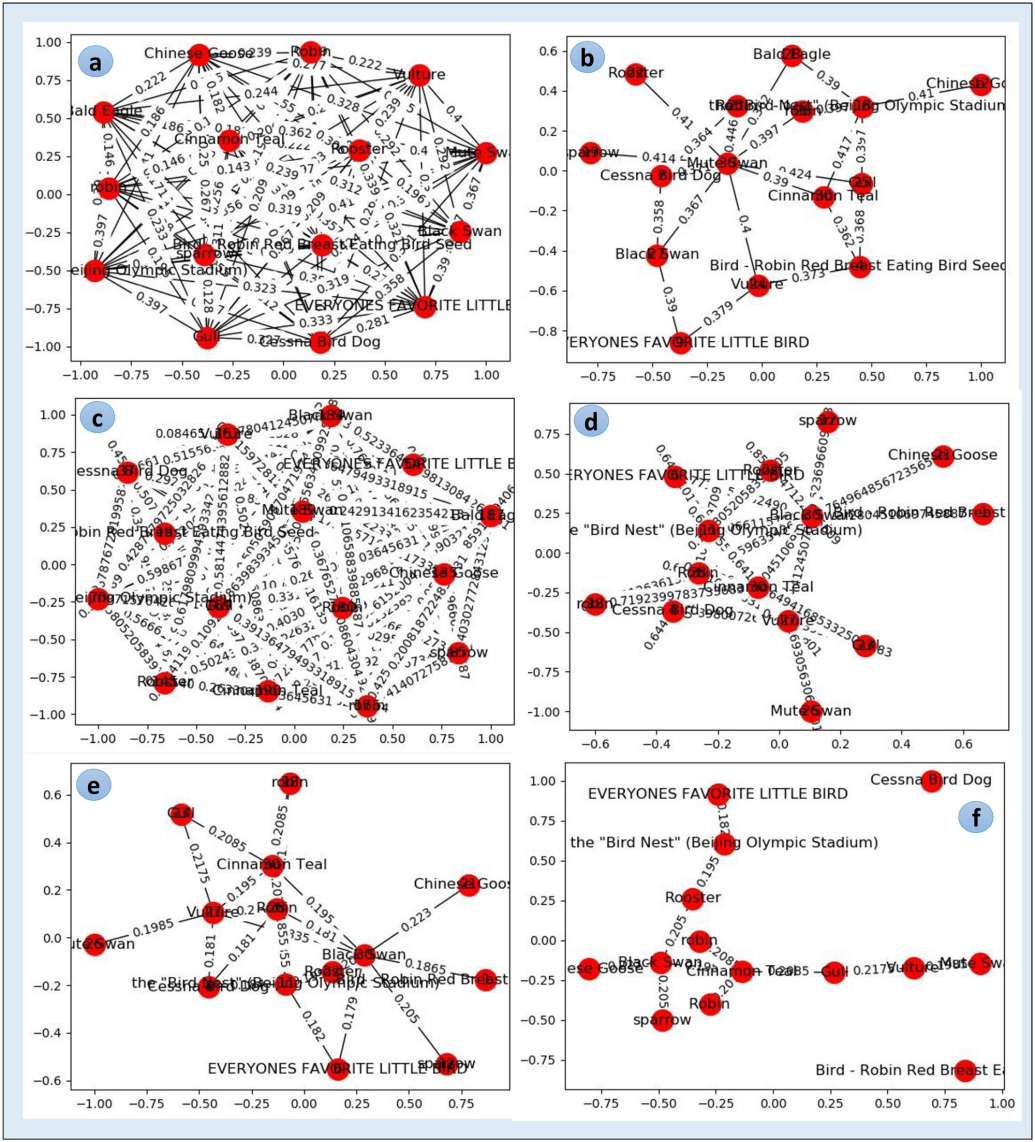
Cluster-graph data model construction over real image results.

In cluster-graph construction, we employed SciPy (https://pypi.org/project/scipy/) and Matplotlib (https://pypi.org/project/matplotlib/) python libraries, both have been used to instantiate the complex graph models. We instantiated the cluster-graph over the images by performing multiple distinct steps. Fig 5 illustrates a real example of cluster-graph construction. We constructed *T*_*MG*_ by computing textual similarity in nodes by calculating Jaccard index of keywords and *V*_*MG*_ by computing distance in nodes by taking Euclidean distance of CEDDs (Fig 5(a) and 5(c)).

$$\begin{array}{c} J(A,B)=\frac{A\cap B}{A\cup B} \end{array}$$ (5)

$$\begin{array}{c} d\left(i,j\right)=\sqrt{{\left(x_{i1}-x_{i2}\right)}^{2}+{\left(y_{j1}-y_{j2}\right)}^{2}} \end{array}$$ (6)

We normalized distance weights of *V*_*MG*_ via min-max method by distributing score in range (0-1) [38]. The min-max is defined as:

$$\begin{array}{c} s_{k}\prime =\frac{s_{k}-min}{max-min} \end{array}$$ (7)

where {*s*_*k*_} are matching scores and *k* = 1, 2, 3, …, *n*. {*s*_*k*_′} are normalized and min-max are estimated matching scores. We minimized *T*_*MG*_ density by removing edges in nodes having textual similarities less than a threshold *γ*_*t*_ defined as:

$$\begin{array}{c} \gamma_{t}=Mean\left(JS\right)+SD\left(JS\right) \end{array}$$ (8)

where *Mean*(*JS*) is mean of Jaccard similarity up to *i* times and *SD*(*JS*) is standard deviation of Jaccard similarity (Fig 5(b)). We reduced *V*_*MG*_ density by eliminating edges in nodes having visual distance greater than a threshold *γ*_*v*_ defined as:

$$\begin{array}{c} \gamma_{v}=Mean\left(ED\right)+SD\left(ED\right) \end{array}$$ (9)

where *Mean*(*ED*) is mean of Euclidean distance up to *i* times and *SD*(*ED*) is the standard deviation of Euclidean distance (Fig 5(d)).

We converted the *V*_*MG*_ distances into similarity weights by subtracting normalized similarity weights from 1. We unified *T*_*MG*_ and *V*_*MG*_ in a multimodal graph *U*_*MG*_ by applying the union criterion. The multimodal edges *E*_*m*_ of *U*_*MG*_ exploits both textual links *E*_*t*_ and visual links *E*_*v*_. The integration of both edges is achieved as:

$$\begin{array}{c} E_{m}=\alpha \left(E_{t}\right)+\beta \left(E_{v}\right) \end{array}$$ (10)

where *α* = *β* = 0.5 (Fig 5(e)). We applied Zahns’ method (*ZAC*) on *U*_*MG*_ to transform it into a cluster-graph. The *ZAC* method computes maximum spanning tree of *U*_*MG*_ using Kruskal’s algorithm and remove edges having weights greater than average weight of their adjacent edges (Fig 5(f)).

### Tool implementation

We implemented an image exploration tool to realize our approach. We must mention that we mainly employed textual information associated with images in retrieval. However, the inverted index also contains pointers to visual descriptors. The SUI has multiple integrated panels (Fig 4). We developed the SUI using multiple open-source libraries, including HTML, CSS, and JavaScript. Particularly, Python libraries, Network-x (https://pypi.org/project/networkx/) and Cystoscape (https://js.cytoscape.org/), are used to visualize the cluster-graphs.

## Results exploration mechanism

The SUI provides an image exploration mechanism. Mainly, the SUI enables various interaction and visualization possibilities by retrieving images, representing results as a cluster-graph, reaching desired results, and enhancing visual perception of results. The SUI allows users to explore images by defining a user interaction paradigm.

### Interaction behavior

**Fig 6:**
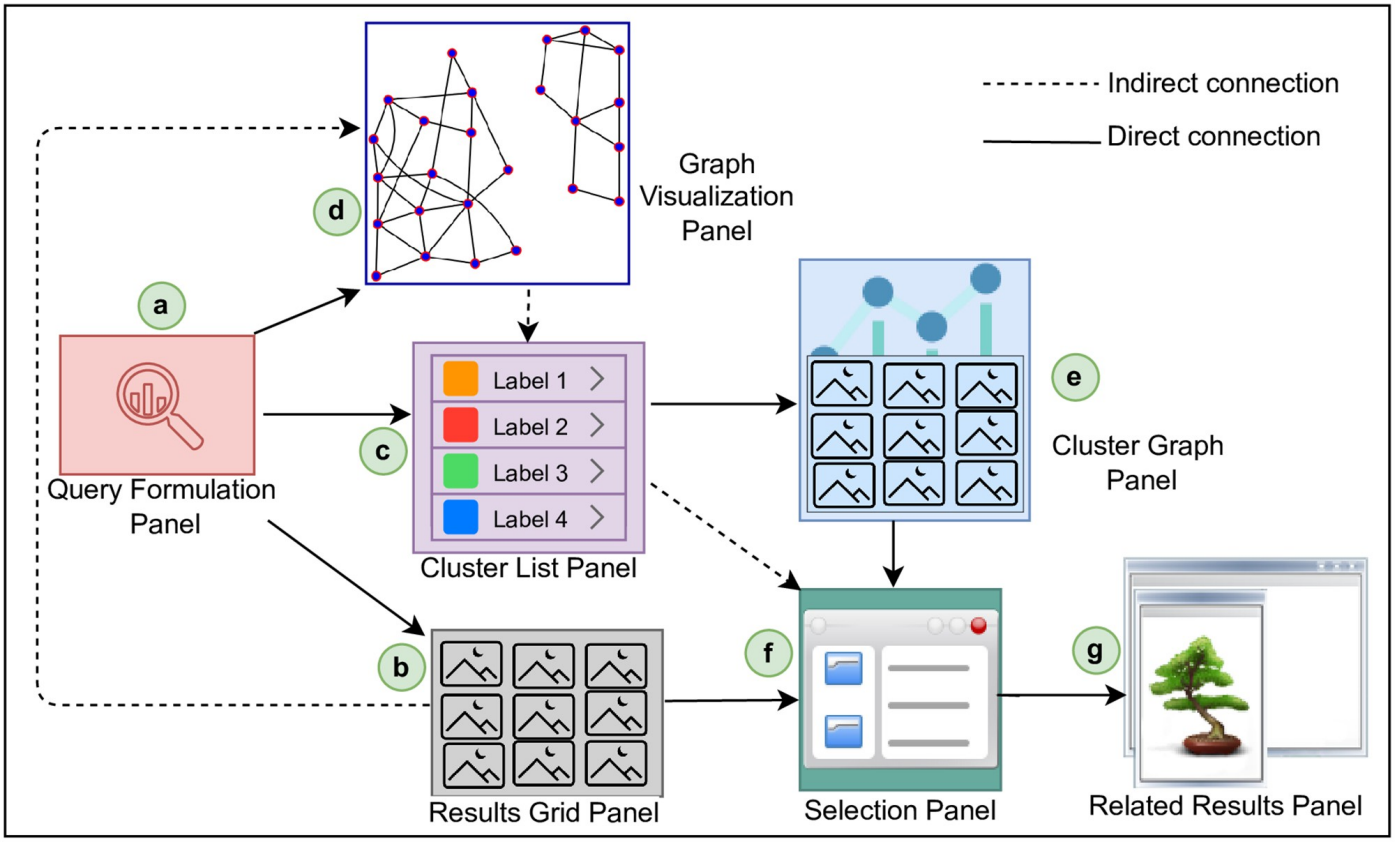
Schematic representation of user interaction paradigm; information need expression (*a*), the exploration initiation (*b*), linear navigation (*c*), and non-linear navigation (*d*).

The interaction activities include information need expression, exploration instantiation, traversing, non-linear navigation, and reachability. The exploration activities are comprehensive and integrated and are provided by various SUI panels. The exploration instantiation enables users to express information needs and perform 2D grid browsing and image lookup via QFP and RGP, respectively (Fig 6(a) & 6(b)). The users can directly navigate SP and view details by selecting an image from RGP. The objective is to overview and look up retrieved images presented in a 2D grid. The selection of images from SP also initializes RRP. It provides a quick lookup of images directly connected in a cluster-graph.

Linear navigation allows users to navigate images sequentially by selecting results from CLP and interacting with a linear list of clusters represented as textual labels. The users can traverse linearly and lookup cluster results. The CLP allows users to explore clusters in a linear way (Fig 6(c)). The selection of a cluster label from CLP populates CGP with related images. Selecting a cluster label from CLP also highlights associated graph visualization in GVP. Non-linear navigation support users to navigate in images visualized via GVP. The users can also view selected images in a 2D grid presented by RGP since RGP is directly connected with GVP. The users can browse and navigate the visualized images of the cluster-graph via GVP (Fig 6(d)). A single click on a node proceeds toward an image preview showing its associated details in a separate window.

The GVP is also directly connected with SP since the users can select a node from GVP, view the related details, and access the corresponding images from their web sources. Images reachability enables the selection of a cluster label from CLP that also presents related images in CGP. However, unlike in traditional 2D grid-based interaction, the users can perform in-depth browsing of images via CGP. The objective is to make results reachable by users via fewer clicks. The users can select any image from CGP and navigate non-linearly to other results in the same cluster. The CLP and CGP are intended to support the users’ exploration activities. The results presented in CGP are also interlinked with SP since a user can look up details of a selected result. The clusters can be chosen from CLP, and images are reachable via CGP.

### Exploration scenario

**Fig 7:**
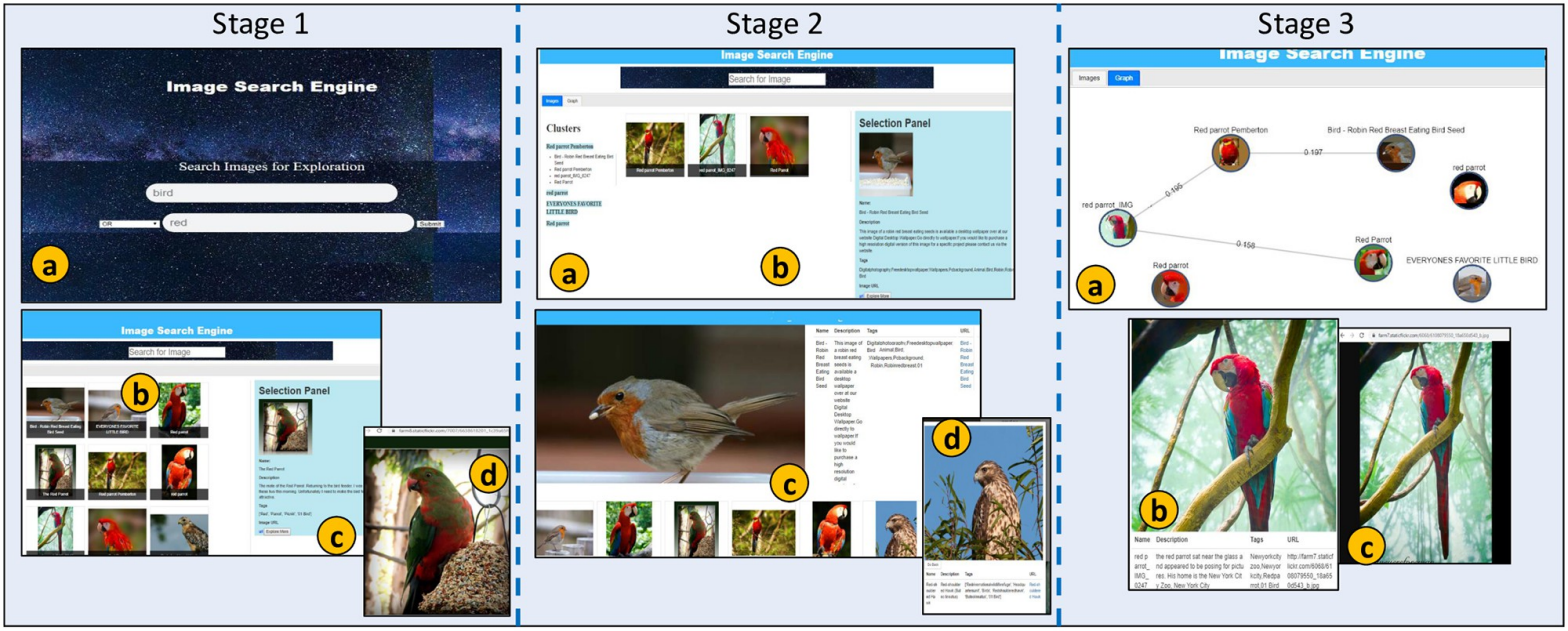
The grid exploration scenario (I) as a query is expressed in QFP (*a*), images results are presented in RGP (*b*), lookup of a selected image in SP (*c*), and access of an image from web source (*d*); The cluster-graph exploration scenario (II) as clusters hierarchy is presented in CLP (*a*), Cluster images presentation in RGP (*b*), lookup of a selected image in SP, exploration of an image in RRP (*c*) and lookup of a selected image in Information Window (*d*); The graph visualization exploration scenario (III) as images visualization in GVP (*a*), lookup of a selected image in Information Window (*b*), and access of an image from web source (*c*), against query ‘Bird’ AND ‘Red Bird’.

The users’ interaction with SUI mainly considers contextual aspects of exploration. It involves reaching results via interactive browsing and visualization options. The SUI enables interaction with images via exploration activities supplemented by dynamic cluster-graph visualization. The users can formulate textual queries, lookup clusters, navigate within clusters, visualize cluster-graph, preview/view details of images, and access them from their actual web sources. It is essential to mention that users can execute alternative exploration scenarios by employing multiple SUI panels, i.e., the users can interact with lists, clusters, grids, graphs, etc., in exploration activities (Fig 7).

#### Grid exploration

The user expresses information needs by giving keywords ‘Bird’ OR ‘Red Bird’ in QFP (Fig 7(I-a)). The query execution populates images in RGP. The user selects ‘Image Tab’ and traverses horizontal-vertical to explore image previews in a 2-D grid presenting image thumbnails. The user selects thumbnail of ‘The Red Parrot’ (Fig 7(I-b)). The SP automatically loads an image, ‘The Red Parrot’, and the user lookup the details as preview, title, description, tags, and URI (Fig 7(I-c)). The user continues exploration by selecting the URI and accessing an image from its actual web source (Fig 7(I-d)). The user interaction with images in RGP is primitive; however, the interaction provides an exploration initiation. The user can continue exploration via multiple SUI panels.

#### Cluster-graph exploration

The CLP presents clusters labeled as ‘Red Parrot Pemberton’, ‘Everyone favorite bird’, and ‘Little Bird’ in extendable lists. The user selects the first cluster label ‘Red Parrot Pemberton’ and views images labeled as ‘Bird-Robin Red…’, ‘Red Parrot Pemberton’, ‘Red Parrot IMG’, and ‘Red Parrot’. The CLP presents labels of clusters and their related images in a hierarchy (Fig 7(II-a)). The clusters’ interaction and connected images are linear; users can look up images in clusters. The user selects ‘Bird-Robin Red…’ from CLP and views its directly connected images, i.e., ‘Red Parrot Pemberton’, ‘red parrot IMG’, and ‘Red Parrot’ in CGP (Fig 7(II-b)).

The user can reach any result by selecting an image from clusters presented in CGP. The user lookup details associated with the chosen image ‘Bird-Robin Red…’ as preview, title, description, tags, and URI via SP. The user selects the option ‘Explore More’ from SP and views similar thumbnails in RRP (Fig 7(II-c)). The user can select CLP, CGP, or RRP results to continue further exploration activities. In this particular scenario, the user selects the image thumbnail ‘Red-shouldered Hawk’ from the RRP and views the details associated with it as preview, title, description, tags, and URI in a separate information window (Fig 7(II-d)).

#### Graph visualization exploration

The user continues exploration by selecting the ‘Graph Tab’. As a response, the GVP visualizes a dynamic cluster-graph of results previewing cluster-graph nodes as image thumbnails along with their corresponding textual titles ‘Red Parrot’, ‘Red Parrot Pemberton’, ‘Bird-Robin Red…’, ‘Red Parrot IMG’, ‘Red Parrot’, ‘The Red Parrot’, and ‘Everyone Favorite little…’. The cluster-graph is divided into four individual accessible clusters (Fig 7(III-a)). The user selects a node titled ‘Red Parrot IMG’ from a cluster of cluster-graph and views the associated details as view, title, description, tags, and URI in a separate information window (Fig 7(III-b)). The interaction with results visualized in a cluster- graph is non-linear. Users can select any node, perform in-depth browsing of cluster-graph, and explore images. The user can navigate within the results visualized in the cluster-graph. The user previews images directly connected with the selected result in RRP as thumbnails (Fig 7(II-c)). The user continues exploration by accessing an image from its actual web source (Fig 7(III-c)).

## Evaluation

We employed (*i*) success-task-completion, (*ii*) time-on-panel & exploration-activity-path, (*iii*) task-difficulty-rating & user-experience, and (*iv*) subjective usability, as measures to evaluate users’ interaction experience in the exploration of image results by employing implemented image results exploration tool. It is important to mention that our approach is SUI-based, and its empirical comparison with other exploration mechanisms is impossible. We declare that the study was for purely academic purposes. The experimentation was conducted in a controlled lab setting, and consent from the participants was verbally taken before conducting human studies. The participants were detailed about the study procedure before experimentation. The research was reviewed and approved by the Advanced Studies and Research Board (ASRB) of Quaid-I-Azam University, Islamabad, Pakistan. The letter describing Authentication and Consent can be accessed from Zenodo repository (https://doi.org/10.5281/zenodo.7027704/).

### Respondents

We recruited 30 respondents for a user study. We considered the sample size reasonable since we referred to Tullis and Stetson concerning the appropriate number of users in such types of evaluations [39]. Demographically, among the 30 respondents, 14 were males, and 16 were females. Their ages varied from 22 to 45 years; the average age was 32 years. The participants had varying information literacy levels. The respondents were further categorized into 10 expert (*EU*_1_-*EU*_10_), 14 average (*AU*_11_-*EU*_24_), and 06 novice (*NU*_25_-*NU*_30_) users. The expert respondents were good at exploration and mostly interacted with web resources by employing advanced search mechanisms. The average respondents interacted with the web to find appropriate information using standard web search mechanisms. The novice respondents were infrequent users who occasionally found relevant information from the web.

### Tasks

We formulated structured and unstructured exploration tasks to evaluate user interaction. The former counterbalances multiple exploration options and highlights diversity in utilizing numerous possibilities in exploration. In the structured exploration task (*ET*_1_), respondents were asked to: (*i*) search keywords ‘Fish’ and ‘Nature’ connected via ‘AND’ operation; (*ii*) discover a graph node presenting ‘Shark’; (*iii*) identify a ‘shark fish’ from a grid, view the associated information, and access an image result from web source; (*iv*) Select the label ‘Peacock Grouper’ from clusters list, scroll screen of images and select a label ‘Pirhana’ from cluster results; (*v*) view the information associated with ‘Pirhana’ and select explore more option, identify the third image from a grid of related images, read the relevant information, and access an image result from web source; (*vi*) select the image ‘Shark’ from the grid, read associated information, and go back to a related set of images. In the unstructured exploration task (*ET*_2_), we presented 02 scenarios to the respondents and requested them to select a scenario of interest, formulate a query, and explore image results. We articulated the scenarios as “formulate a query for red birds and explore image results” and “formulate a query for green reptiles and explore image results” using the given exploration tool.

### Instruments

We evaluated the usability of our approach via multiple instruments. The recent literate is evident of successful-task-completion and time-on-panel measures employment in usability evaluation [12, 40]. We also utilized similar measures to evaluate user interaction behavior. We selected a set of standard questionnaires, i.e., task-difficulty-rating and user-experience [41] to analyze the user experience. We further employed state-of-the-art instruments, which are the System Usability Scale (SUS) [42] and Computer System Usability Questionnaire (CSUQ) [43] in subjective usability evaluation. In addition to this, we also recorded and analyzed users’ exploration activities. Particularly we employed the users’ activity diagram and heat maps to analyze users’ information-seeking behavior in image results exploration [44].

### Procedure

The evaluation was conducted in a peaceful ambiance. The image exploration tool was deployed on a workstation with peripheral devices, including a mouse, keyboard, and monitor. A screen recorder was installed to capture the respondents’ interactions with the tool. Before the assessment, respondents were briefly introduced to a user study and image exploration tool. The respondents were also shown a 5 minute video tutorial to elaborate on the exploration mechanism. The respondents were encouraged to query about the tool and related exploration mechanisms. Once the preliminary steps were concluded and after getting demographic details, the respondents were asked to sit on a comfortable chair and perform exploration tasks via our image exploration tool. At the end of the exploration tasks, the respondents were given a set of questionnaires to provide their interaction, seeking, and exploration feedback.

### Data availability statement

The research study data containing evaluation data and results; extracted and transformed dataset used in the evaluation; I-Search actual multimodal dataset; Image search tool; and installation instructions in a readme file, supporting the findings of this research study are openly available in Zenodo repository (https://doi.org/10.5281/zenodo.6388246).

## Results and discussion

### Successful-task-completion

**Fig 8:**
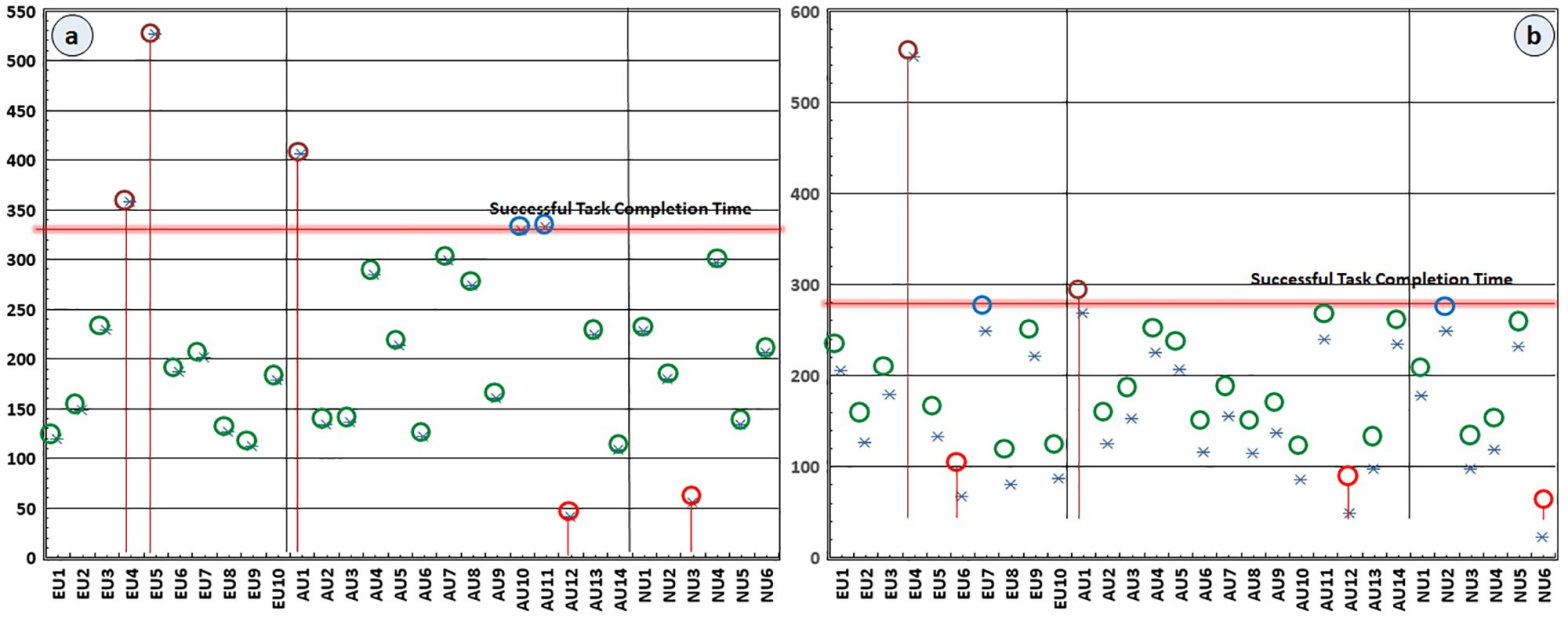
Respondents task completion time of exploration tasks ET_1_ (a) and ET_1_ (b).

We recruited 5 respondents from the expert, average, and novice user groups to compute the successful-task completion time. We requested them to learn the tool and associated exploration mechanism via (training manual and practice). We asked them to complete the exploration tasks (ET_1_ and ET_2_). We recorded their exploration activities and computed the successful task completion thresholds STC_1_ and STC_2_ of ET_1_ and ET_2_, respectively. We computed STC_1_ and STC_2_ by taking the sum of mean task completion time and standard deviation of ET_1_ and ET_2_, respectively. The STC_1_ of ET_1_ and STC_2_ of T_2_ are 550 sec and 330 sec, respectively. We also recorded the task completion time TCT ${}_{ET_{1i}}$ and TCT ${}_{ET_{2i}}$ of ET_1_ and ET_2_ of each user, respectively. TCT of individual users are summarized in Fig 8(a) and 8(b).

The TCT of individual users above the STC_1_ and STC_2_ encircled maroon indicate that respondents failed to complete the exploration tasks within time limits. The respondents below STC_1_ and STC_2_ encircled green demonstrate that they efficiently completed the exploration tasks within time limits. The respondents at borderline encircled blue indicate that they just completed the exploration tasks successfully within time limits with few difficulties. The respondents encircled red mean that they faced problems in the performance of exploration tasks and voluntarily withdrew before the expiry of time limits.

Fig 8(a) and 8(b) highlight that most respondents completed the assigned exploration tasks within given time limits. The average successful task completion time is within STC_1_ and STC_2_ limits of ET_1_ and ET_2_, respectively. The successful task completion reaches 83.33%; it employs that exploration task type (structured and unstructured) cannot significantly affect the respondents’ exploration activities. The successful-task completion time of different respondents across the various user groups is also uniform. The successfully-task completion time of expert, average, and novice respondent groups in exploration tasks is uniform and reaches 80%, 93.33%, and 90%, respectively.

As emerges from Fig 8(a) and 8(b) that 10% respondents completed ET_1_ fully or partially after passing STC_1_ limit and 6.67% respondents completed ET_2_ fully or partially after passing STC_2_ limit. In ET_1_ and ET_2_, 6.67% and 10% respondents failed to complete the tasks in STC_1_ and STC_2_ limits, respectively. In both exploration tasks, 6.67% respondents were at borderline; however, they completed the exploration tasks with difficulties. The successful-task completion time across the different respondent groups (experts, average, and novices) is uniform, indicating that the diversity in respondents and exploration tasks does not affect task completion.

### Time-on-panel & user-exploration activity

**Fig 9:**
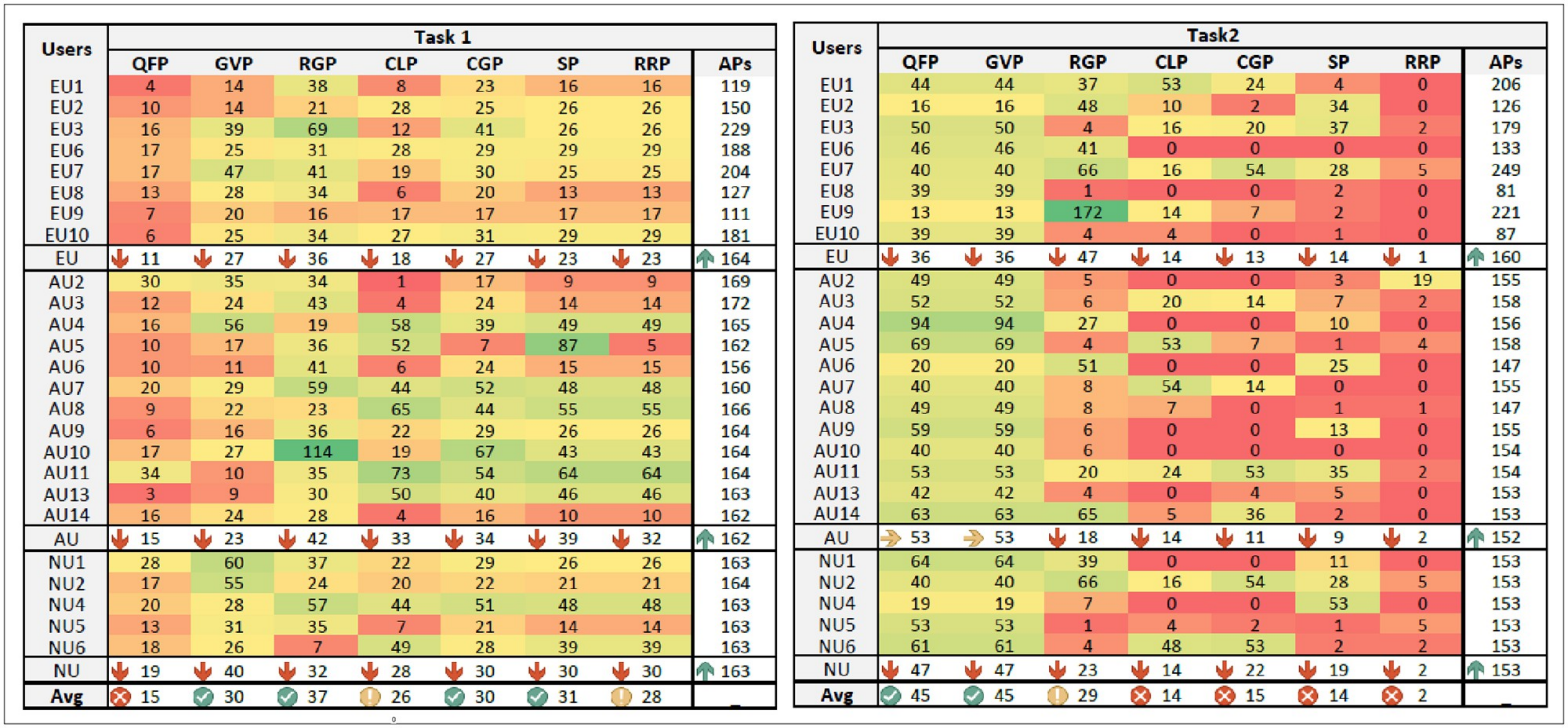
Respondents time-on-panel in the performance of exploration tasks ET_1_ and ET_2_, (Where AP is the average precision of panels and Avg is the overall average).

We recorded the user interactions and analyzed the time-on-panel spent by respondents. We considered only the respondents who completed tasks ET_1_ and ET_2_ within STC_1_ and STC_2_ limits, respectively, since they completed the exploration task in a successful time. The heat maps demonstrate the time on-panel spent by respondents to perform exploration tasks. The heat maps highlight time-on-panel via different variations (light to dark) of primary hue colors (i.e., red and green) and decimal values in seconds. The darkest green and red shades show respondents’ maximum and minimum panels utilization in time, respectively. The yellow color demonstrates the average utilization of panels. The different hue variations lie from red to green with changing gradients (darkest red to darkest green). The ranges (smallest to largest) interpret variation in time-on-panel by respondents. Fig 9 depicts the heat maps of ET_1_ and ET_2_, respectively.

As it emerges from Fig 9 that overall, respondents in different groups spent minimum time on QFP (represented by various shades of red or yellow color). Our investigation reveals that respondents focused on the exploration activities instead of query reformulation and refinement. The utilization of GVP, RGP, CGP, and SP is high. The respondents mainly employed GVP, RGP, and CGP in exploration and SP to access actual content. Comparatively, CLP and RRP are less used panels in different user groups. CLP involves linear interaction, and RRP visualizes a few additional results. Despite that, the utilization of multiple SUI panels in exploration is marginal since exploration requires the execution of a structured exploration task where exploration activities are well-defined.

Fig 9 also interprets few interesting results. E.g., the yellow color shows that AU_2_ spent (30 sec), and darker red depicts that EU_1_ spent (4 sec) on QFP (lowest time). The GVP (represented by light green color, utilized for 60 sec), RGP (represented by dark green color, utilized for 114 sec), CGP (represented by light green color, utilized for 67 sec), and SP (represented by green color, utilized for 87 sec) and RRP (represented by green color, utilized for 64 sec) are highly utilized panels by NU_1_, AU_10_, AU_11_, and AU_5_, respectively. The respondents’ AU_1_ and AU_5_, represented by dark red color, utilized CLP for 1 and 5 sec, respectively. The differences in average time spent to complete the exploration task (by employing different panels) by the expert, average, and novice user groups are marginal.

As it emerges from Fig 9, the overall number of respondents in different user groups spent minimum time on RRP (represented by various shades of red color). Since RRP gives additional exploration options, most respondents cannot interact with RRP since the time-on-panel is 0 sec (represented by the darkest red color). Respondents were not interested in employing it in unstructured exploration tasks. The utilization of CLP and CGP is comparatively low, as multiple respondents cannot interact with the panels since the time-on-panel is 0 sec (represented by the darkest red color). Mainly the time-on-panel is designated via different shades of red (interpreting the low utilization of panels). Alternatively, almost in all user groups, the respondents preferred to employ GVP and RGP. The investigation reveals that the respondents take more interest in non-linear exploration and visualization.

The respondents considered graph-based visualization more effective in unrestricted exploration activities. In addition to this, they are also interested in exploring via 2-D grids. The utilization of SP is low (mainly represented via different shades of red color) since the comprehensive presentation and visualization options satisfied the information needs without accessing content from their actual web sources. It concludes that in non-linear image representations, the graphical layout is more efficient and user-friendly than a 2-D grid. The difference in average time spent to complete the exploration task (by employing different panels) by the expert, average, and novice users are marginal.

**Fig 10:**
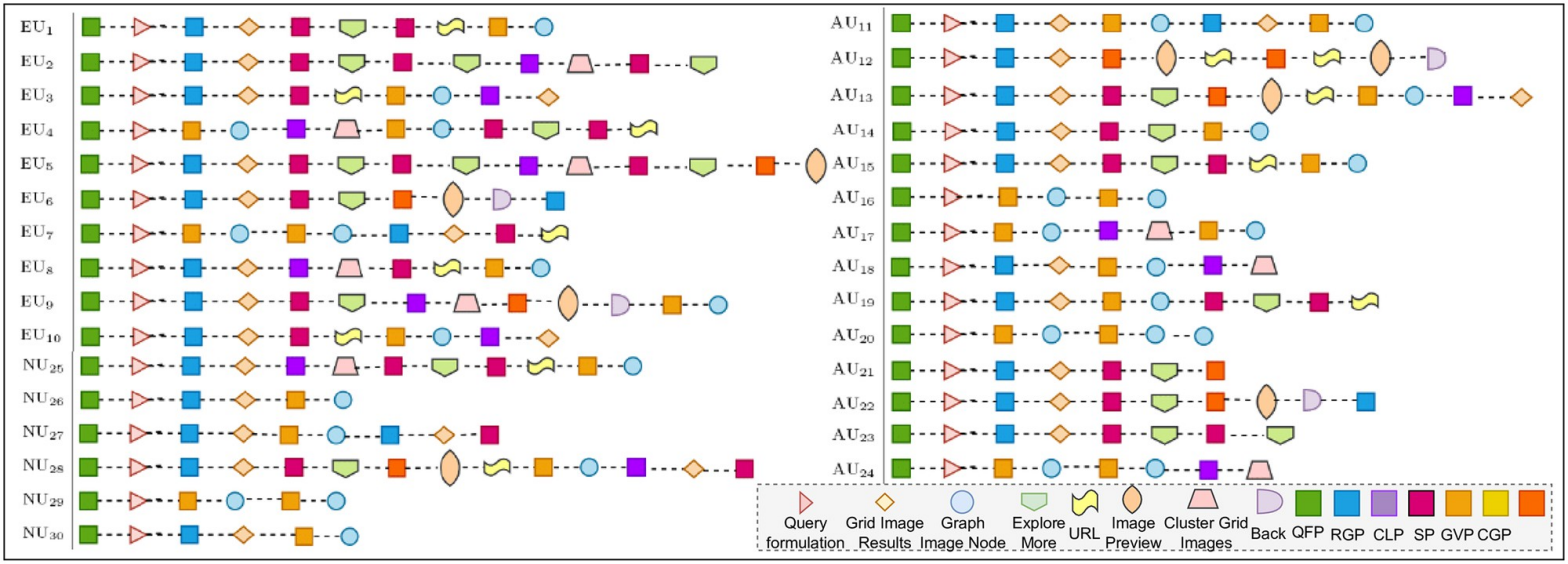
Respondents exploration activity in unstructured task ET_2_.

We continued the analysis by considering the respondents’ exploration activities. We only consider exploration task ET_2_ since the exploration task is unstructured, and respondents were not restricted to select particular panels. They were allowed to select their desired SUI panels to complete exploration tasks. Fig 10 depicts the respondents interaction activities via multiple panels. As it emerges from Fig 10 that respondents had diverse exploration patterns via multiple SUI panels. However, some respondents had similar exploration actions to satisfy their information needs. The exploration patterns depicted respondents performing an exploratory search since they connected related chunks collected from multiple SUI panels.

In Fig 10, the square boxes filled with different colors represent SUI panels. The multiple shapes represent other choices and actions employed by respondents in exploration. The EU_5_ had an overall most extended exploration pattern. However, it lacks the utilization of all panels in prospecting. The RGP and GVP were overall most utilized panels by AU_24_ and AU_12_, respectively. The shortest exploration path was recorded for NU_6_, using only RGP and GVP; the respondent could not complete the exploration tasks. The second-longest track was recorded for NU_28_, practicing more exploration activities than EU_5_. 53.33% respondents were interested in detailed exploration, browsing, and visualization; therefore, they utilized the “Explore more” option. Overall the search paths demonstrated that respondents were interested in exploration non-linearly and graphically. The analysis of exploration activity reveals that browsing activities minimize query formulation and reformulation. Furthermore, the exploration cycle continues iteratively across the different SUI panels.

### Task-difficulty-rating & user-experience

The task difficulty rating is an instrument to measure difficulty associated with user tasks [45]. The instrument contains items to rate the difficulty of a task via a 6-points Likert scale response. The lowest rating scores depict lower task difficulty and vice versa. Alternatively, the user experience is an instrument to measure the user experience associated with using tools in task performance [46]. The instrument contains 5-items to judge the different perspectives of the user task experiences, i.e., ‘familiarity’, ‘accomplishment’, ‘confidence’, ‘completion’, and ‘flexibility’ via a 5-points Likert scale. In a particular perspective, the highest Likert scale position depicts a good user experience and vice versa. Table 1 highlights the task-difficulty-rating and user-experience results.

**Table 1:** Respondents task-difficulty-ratings & user-experience evaluation.

Test	Task Difficulty Rating			User Task Experience		
R.No.	Diff-Rating	Score-Value	Avg	Avg User-Exp	Score-Value	Avg
*EU*_1_	1	17	24	3.4	68	79
*EU*_2_	4	67		4.8	96	
*EU*_3_	3	50		3.8	76	
*EU*_4_	1	17		4.4	88	
*EU*_5_	3	50		4	80	
*EU*_6_	1	17		3.4	68	
*EU*_7_	4	67		3.8	76	
*EU*_8_	3	50		3.6	72	
*EU*_9_	0	0		4.4	88	
*EU*_10_	4	67		3.6	72	
*AU*_14_	1	17	40	3	60	78
*AU*_12_	1	17		4.4	88	
*AU*_13_	1	17		4	80	
*AU*_14_	2	33		4.6	92	
*AU*_15_	1	17		4	80	
*AU*_16_	4	67		3.4	68	
*AU*_17_	0	0		3.2	64	
*AU*_18_	1	17		4.8	96	
*AU*_19_	0	0		3	60	
*AU*_20_	2	33		3	60	
*AU*_21_	2	33		4.4	88	
*AU*_22_	3	50		5	100	
*AU*_23_	1	17		4	80	
*AU*_24_	1	17		4.4	88	
*NU*_25_	1	17	19	4	80	84
*NU*_26_	2	33		4.4	88	
*NU*_27_	0	0		4.4	88	
*NU*_28_	2	33		3.4	68	
*NU*_29_	2	33		4.4	88	
*NU*_30_	0	0		4.6	92	

Table 1 shows that task-difficulty-rating score values of novice and expert user groups are 19% and 24%, respectively. It is 40% in the average user group because average respondents compared the task difficulty of exploration activities with other online exploration mechanisms. However, the experts critically analyzed and compared the exploration mechanism with others and considered it convenient to explore the results. The task-difficulty-rating score values of all user groups are acceptable. The overall task-difficulty-rating score is (28%) with std dev of 11%, depicting that the exploration activity is easy to perform. Table 1 presents that all user groups found it is a positive experience. The user-experience score values are 84%, 78%, and 79% in novice, average, and expert user groups, respectively. The overall user experience is also favorable since the score value is 80% with std dev of 11%. Overall, the exploration mechanism has passed task-difficult-rating and user-experience usability tests.

### Subjective usability

The SUS is a reliable tool to evaluate the users’ satisfaction associated with interactive systems [47, 48]. The SUS tool contains 10-items to judge overall users’ satisfaction using interactive systems via a 5-points Likert-scale response. Odd-numbered items measured the positive aspects of the SUS questionnaire, and even-numbered items measured the negative aspect of interactive systems. The SUS evaluation yields a score value of (1–100) to depict the overall usability of the interactive system under investigation. Table 2 present the SUS results. In SUS evaluation, the raw score value of items is firstly summed together since the contribution of each item is from 0 to 4. Each odd-numbered item’s raw score value is computed by subtracting 1 from the item contribution. Alternatively, the raw score value of each even-numbered item computation involves its contribution subtraction from 5. The product of summed raw score values and 2.5 gives the overall SUS score value. Following the procedure, the SUS score value is 76.83% with an std dev of 10.99%. The standard SUS scale given in [49] ranked our computed SUS score value as ‘acceptable’ and ‘good’. It means that the provided interaction has passed the usability test, and users are satisfied with our proposed image results exploration mechanism.

**Table 2:** Respondents SUS evaluation scores.

R.No.	1	2	3	4	5	6	7	8	9	10	Score-Value	Avg
*EU*_1_	1	4	2	5	2	4	2	5	1	3	72.5	73
*EU*_2_	3	4	3	4	3	4	3	4	3	4	87.5	
*EU*_3_	2	3	0	3	2	3	2	3	2	4	60	
*EU*_4_	2	3	-1	3	1	3	3	3	1	5	57.5	
*EU*_5_	2	5	3	2	3	3	2	3	3	5	77.5	
*EU*_6_	2	1	2	3	1	2	2	3	1	4	52.5	
*EU*_7_	2	4	0	4	2	4	3	5	3	1	70	
*EU*_8_	2	4	3	4	3	5	2	4	3	5	87.5	
*EU*_9_	1	4	3	5	2	5	3	3	2	4	80	
*EU*_10_	2	5	2	4	3	4	2	5	3	4	85	
*AU*_11_	2	4	2	2	2	5	2	3	0	2	60	82.5
*AU*_12_	3	5	2	5	3	5	1	3	2	5	85	
*AU*_13_	2	4	2	2	2	4	2	4	0	2	60	
*AU*_14_	2	3	3	2	1	3	0	4	1	3	55	
*AU*_15_	3	4	1	3	3	4	3	4	3	3	77.5	
*AU*_16_	3	5	2	5	-1	4	3	5	2	5	82.5	
*AU*_17_	3	5	3	5	2	5	3	5	3	5	97.5	
*AU*_18_	2	2	2	4	2	4	1	3	2	4	165	
*AU*_19_	1	4	0	5	1	4	3	3	2	4	67.5	
*AU*_10_	2	5	3	5	3	5	2	5	3	2	87.5	
*AU*_11_	2	4	2	4	2	4	2	4	1	4	72.5	
*AU*_12_	3	4	2	4	2	5	3	5	3	2	82.5	
*AU*_13_	2	5	3	5	3	5	2	5	3	4	92.5	
*AU*_14_	1	4	3	4	2	5	1	5	1	2	70	
*NU*_25_	2	5	2	4	0	4	1	4	1	4	67.5	75
*NU*_26_	3	4	3	5	-1	4	3	1	3	5	75	
*NU*_27_	2	1	0	5	2	5	3	3	3	5	72.5	
*NU*_28_	2	3	0	4	2	5	3	4	3	5	77.5	
*NU*_29_	2	4	2	5	2	5	2	3	2	4	77.5	
*NU*_30_	3	4	-1	5	3	5	3	2	3	5	80	

The CSUQ is a popular tool usually employed to evaluate the usability of a broad range of interactive systems [50]. The CSUQ contains 19-items to judge different aspects of interactive systems via a 7-points Likert scale. The CSUQ measures system usefulness (items 1–8), information quality (items 9–15), and interface quality (items 16–18) associated with interactive systems, along with overall usability. Table 3 present the CSUQ experimental results. The comprehensive user usability assessment is excellent (the score value is 82.98% and std dev is 1.23%). The respondents also appreciated the systems’ usefulness (score value is 83.73% and std dev is 1.11%), information quality (score value is 85.71% and std dev is 1.08%), and interface quality (score value is 82.98% and std dev is 1.23%). The experimental results also show that the difference in score values associated with system usefulness, information quality, and interface quality is marginal. Our investigation reveals that overall usability and various usability dimensions are equally popular among all user groups.

**Table 3:** Respondents CUSQ evaluation scores.

R.No.	Overall	Usefulness	Info. Qua.	Int. Qua.	Score-Value	Avg
*EU*_1_	1.25	2.13	2	1.25	23.71	78.4
*EU*_2_	6.75	6.88	7	6.75	97.86	
*EU*_3_	5.25	5.13	5	5.25	73.71	
*EU*_4_	5.75	6.63	6	5.75	86.14	
*EU*_5_	6	5.25	6	6	83	
*EU*_6_	5.75	5.13	5	5.75	77.29	
*EU*_7_	6	6.25	7	6	90.14	
*EU*_8_	5.25	5.63	6	5.25	79	
*EU*_9_	6.5	6.25	7	6.5	93.71	
*EU*_10_	5	6.13	6	5	79	
*AU*_11_	5.25	3.38	6	5.25	71	86
*AU*_12_	6.5	5.88	7	6.5	92.43	
*AU*_13_	3.75	5	4	3.75	59	
*AU*_14_	5	5.63	5	5	73.71	
*AU*_15_	6	6.13	6	6	86.14	
*AU*_16_	6.75	6.88	7	6.75	97.86	
*AU*_17_	7	7	7	7	100	
*AU*_18_	7	6.25	6	7	93.71	
*AU*_19_	5.25	7	6	5.25	84	
*AU*_20_	7	6.88	6	7	96	
*AU*_21_	5	4.5	5	5	69.71	
*AU*_22_	7	6.38	7	7	97.86	
*AU*_23_	6.75	6.38	7	6.75	96	
*AU*_24_	6	6.25	6	6	86.57	
*NU*_25_	4.25	6	6	4.25	73.29	88
*NU*_26_	6.75	6.25	7	6.75	95.57	
*NU*_27_	6.25	6.75	7	6.25	93.71	
*NU*_28_	5.5	4.63	6	5.5	77.29	
*NU*_29_	6.75	6.25	6	6.75	92	
*NU*_30_	7	7	6	7	96.43	

## Comparison & discussion

The recent research refers to various image results exploration tools. In addition, a wide variety of online tools are also available over the web. The image search and exploration tools usually employ a particular SUI design approach to enhance the user’s experience. We extracted essential parameters and attribute values from the literature to provide a comprehensive parametric comparison of our approach with the existing online and state-of-the-art image results exploration tools discussed in the literature. The extracted parameters mainly emphasize the two aspects, i.e., SUI design and human behavior. The former includes representation, presentation, and visualization of search results. The latter address the human behavioral aspects in image results exploration. Table 4) exhibits the conformance and unavailability of parameters in image results search and exploration tools as ‘✓’ and ‘-’, respectively.

**Table 4:** Theoretical comparison of online image search tools; state-of-the-art search and exploration tools discussed in the literature and our proposed SUI approach.

Parameters		Online Image SUIs										Literature based Image SUIs							
Attributes	Values	Google	Yahoo	Shutter-stock	Flickr	Photo-bucket	Bing	Pinterest	Getty	Yandex	Duck-DuckGo	MIRRE	M^2^IS	FACETS	Media-Finder	Visual-Islands	Image Search Interface	Faeric-World	SUI Approach
Query	Textual	✓	✓	✓	✓	✓	✓	✓	✓	✓	✓	✓	✓	✓	✓	✓	✓	✓	✓
Modality	Visual	-	-	-	-	-	-	-	-	-	-	-	-	-	-	-	-	-	-
Grid View	Static	✓	✓	-	✓	-	✓	✓	✓	-	✓	-	-	-	✓	-	✓	-	-
	Dynamic	-	-	✓	-	-	-	-	-	✓	-	✓	✓	-	-	✓	-	-	✓
Grid View	Title	✓	-	-	✓	-	-	-	✓	✓	✓	✓	✓	-	-	-	-	-	✓
Details	Details	✓	-	-	✓	-	✓	✓	✓	✓	-	-	✓	-	-	-	-	-	✓
Clusters	Hirerachical	-	-	-	-	-	-	-	-	-	-	-	-	-	-	-	✓	✓	✓
	Non-Hirearchical	✓	-	-	✓	-	✓	-	-	-	-	-	-	✓	-	✓	-	-	-
Cluster	Highest Similiairty	-	-	-	-	-	-	-	-	-	-	-	-	✓	-	-	-	-	-
Fingerprint	Highest Semantics	-	-	-	-	-	-	-	-	-	-	-	-	-	-	-	-	-	-
Non-linear	Tree	-	-	-	-	-	-	-	-	-	-	-	-	-	-	-	✓	-	✓
Visualization	Graphs	-	-	-	-	-	-	-	-	-	-	✓	✓	✓	-	-	-	✓	✓
Non-linear	Navigable	-	-	-	-	-	-	-	-	-	-	✓	-	✓	-	-	✓	-	✓
Image	Non-Navigable	-	-	-	-	-	-	-	-	-	-	-	✓	-	-	-	-	-	-
Info Access	Actual Sources	✓	-	-	-	✓	✓	✓	-	✓	✓	✓	✓	-	-	-	-	-	✓
	Provided Data	✓	✓	✓	✓	-	-	✓	-	✓	✓	-	✓	✓	✓	✓	✓	✓	✓
Panels	Integrated	✓	-	✓	-	-	✓	✓	-	✓	-	✓	✓	-	-	-	-	-	✓
	Non-Integrated	-	-	-	-	-	-	-	-	-	-	-	-	-	-	-	-	-	-
Image	Ranked	✓	✓	✓	✓	✓	✓	✓	✓	✓	✓	✓	✓	-	✓		✓	✓	✓
Results	Un-Ranked	-	-	-	-	-	-	-	✓	-	-	-	-	✓	-	✓	-	-	-
Image	Similairty	✓	✓	✓	✓	✓	✓	✓	-	✓	✓	✓	✓	-	-	-	✓	✓	✓
Results	Features	-	-	-	-	-	-	-	-	-	-	-	-	✓	✓	-	-	-	-
Preview	Textual	-	-	✓	-	-	-	-	-	✓	✓	✓	✓	-	✓	-	-	-	✓
Modality	Visual	-	-	✓	-	-	-	-	-	✓	✓	✓	✓	-	✓	-	-	-	✓
Graph Nodes	Text Nodes	-	-	-	-	-	-	-	-	-	-	✓	✓	✓	-	-	-	✓	-
Visualization	Image Nodes	-	-	-	-	-	-	-	-	-	-	-	-	-	-	-	-	-	✓
Graph Edges	Similarity Edges	-	-	-	-	-	-	-	-	-	-	✓	✓	-	-	-	-	✓	✓
Visualization	Semantics Edges	-	-	-	-	-	-	-	-	-	-	✓	-	✓	-	-	-	-	-
Browsing	Simple	✓	✓	✓	✓	✓	✓	✓	✓	✓	✓	✓	✓	✓	✓	✓	✓	✓	✓
	In-depth	-	-	-	-	-	-	-	-	-	-	✓	✓	✓	-	-	-	✓	✓
Scrolling	Finite	✓	✓	✓	✓	✓	✓	✓	✓	✓	✓	✓	✓	✓	✓	✓	✓	✓	✓
	Infinite	-	-	-	-	-	-	-	-	-	-	-	-	-	-	-	-	-	-
Navigation	Sequential	✓	✓	✓	✓	✓	✓	✓	✓	✓	✓	✓	✓	✓	-	-	✓	✓	✓
	Non-Sequential	-	-	-	-	-	-	-	-	-	-	✓	✓	✓	-	-	✓	✓	✓
Search Form	Lookup	✓	✓	✓	✓	✓	✓	✓	✓	✓	✓	✓	✓	✓	-	-	-	✓	✓
	Exploratory	-	-	-	-	-	-	-	-	-	-	✓	✓	✓	✓	✓	✓	✓	✓

As it emerges from Table 4 that parameters linked with the SUI define query modality as query formulation that can be expressed via textual or visual queries. Generally, the retrieved images have been presented in 2-D grids, allowing horizontal and vertical scrolling. The static grid displays the fixed images, while the dynamic grid permits the users to browse the individual image results accordingly. The grid view presents image titles or details (tags, publisher, category, and date), indicating that users can be interested in hovering over the search results to perceive their details. Clusters are similarity-based image groups that contain search results that are further organized in hierarchies. The users can explore the sub-graphs belonging to clusters in their exploration activities.

As it emerges from Table 4 that parameters linked with the SUI define query modality as query formulation that can be expressed via textual or visual queries. Generally, the retrieved images have been presented in 2-D grids, allowing horizontal and vertical scrolling. The static grid displays the fixed images, while the dynamic grid permits the users to browse the individual image results accordingly. The grid view presents image titles or details (tags, publisher, category, and date), indicating that users can be interested in hovering over the search results to perceive their details. Clusters are similarity-based image groups that contain search results that are further organized in hierarchies. The users can explore the sub-graphs belonging to clusters in their exploration activities.

The human interaction parameters allow users to initiate search activities by expressing their information needs. The search activities involve multiple integrated lookup and exploration activities. The users can scroll and browse the results iteratively; however, grid layouts lack exploration in terms of in-depth browsing. Therefore, non-linear visualizations may usually support in-depth browsing of the image results. The navigation can be classified as sequential and non-sequential. The linear presentations provide sequential navigation within the image results. Alternatively, non-linear exploration may be possible via trees and graphs that can give irregular searching patterns known as non-sequential exploration.

The comparative table concludes that few image search and exploration tools provide in-depth browsing, non-linear visualization, and navigation. The proposed SUI approach depicts that our image exploration tool covers all significant parameters extracted from the literature. It includes query formulation, fingerprint clusters, hierarchical trees, and navigable graphs with image nodes that initiate in-depth browsing, scrolling, and navigation (sequential and non-sequential) activities. Our Image preview exploits multimodality; however, users can also access information from the dataset and actual sources. Our investigation reveals that the proposed image exploration tool is practical, effective, and user-friendly in seeking activities. It is crucial to mention that our approach is multimodal and exploits multimodal relationships to visualize image results. To the best of our knowledge, a similar exploration tool has not been presented before that follows the most significant exploration parameters provided by web image search engines and image exploration tools discussed in the literature.

## Conclusions and future research

Despite the interactive presentation and visualization enabling various exploration activities to satisfy users’ information needs, web image exploration remains unaddressed. Our research describes an SUI-based approach to provide a non-linear and multimodal exploration of image results in an integrated and usable way. In particular, our approach suggests using a graph-cluster data model exploiting the textual and visual modalities enabling non-linear and multimodal representation of image results. The SUI presents and visualizes image results via interactive graph visualization; the objective is to offer image results exploration, browsing, and navigation within the selected clusters of the image results graph.

We evaluated the proposed SUI-based approach via multiple usability tests to analyze user behavior, experience, and interaction. Time analysis employed successful-task completion and time on the panel to assess respondents’ time spent on the tool while performing exploration activities. The overall successful-task measure indicates that irrespective of category selection, the task was a success with 83.33%, and the time-on-panel and user exploration activity pattern disclosed that, on average, the utilization of graphical visualization was high as compared to other exploration options. In addition, user activity-based search paths divulged respondents interested in exploring web images non-linearly and graphically via our SUI-based exploration approach. The task-difficulty-rating and task experience assessed the respondents’ difficulties while exploring the search results.

The investigation of task difficulty revealed that all users faced acceptable challenges with an average 28% difficulty rating score. In addition, the overall user experience was excellent (above 80%). Therefore, the exploration mechanism has passed both usability tests. The standard SUS and CUSQ measured user satisfaction and system usability, respectively. The SUS instrument outcome showed that the overall score was 76.83%, which ranked our tool as ‘acceptable and good’ on a standard scale. The overall usability score computed via the CUSQ instrument was 83%. The usability scores of SUS and CUSQ conveyed that our approach is highly satisfactory and usable. The overall evaluation revealed that the proposed SUI provided a practical and adequate exploration of web image results.

We will investigate the visual exploration of multimedia content, particularly in a Big Data context in the future. We will investigate mechanisms to discover visual resources, deep learning approaches, and advanced presentation and visualization schemes. We intend to address reachability issues and comparatively less complex non-linear data models to provide effectiveness in image exploration activities.
